# Silver Conductive Threads-Based Embroidered Electrodes on Textiles as Moisture Sensors for Fluid Detection in Biomedical Applications

**DOI:** 10.3390/ma14247813

**Published:** 2021-12-17

**Authors:** Saima Qureshi, Goran M. Stojanović, Mitar Simić, Varun Jeoti, Najeebullah Lashari, Farooq Sher

**Affiliations:** 1 Faculty of Technical Sciences, University of Novi Sad, T. Dositeja Obradovića 6, 21000 Novi Sad, Serbia; sgoran@uns.ac.rs (G.M.S.); mitar.simic@uns.ac.rs (M.S.); varunjeoti@uns.ac.rs (V.J.); 2 Department of Petroleum Engineering, Universiti Teknologi PETRONAS, Seri Iskandar 32610, Perak Darul Ridzuan, Malaysia; najeebullah.lashari@duet.edu.pk; 3 Department of Engineering, School of Science and Technology, Nottingham Trent University, Nottingham NG11 8NS, UK

**Keywords:** sustainable materials, embroidered electrodes, textile sensor, moisture impedance and biomedical

## Abstract

Wearable sensors have become part of our daily life for health monitoring. The detection of moisture content is critical for many applications. In the present research, textile-based embroidered sensors were developed that can be integrated with a bandage for wound management purposes. The sensor comprised an interdigitated electrode embroidered on a cotton substrate with silver-tech 150 and HC 12 threads, respectively, that have silver coated continuous filaments and 100% polyamide with silver-plated yarn. The said sensor is a capacitive sensor with some leakage. The change in the dielectric constant of the substrate as a result of moisture affects the value of capacitance and, thus, the admittance of the sensor. The moisture sensor’s operation is verified by measuring its admittance at 1 MHz and the change in moisture level (1–50) µL. It is observed that the sensitivity of both sensors is comparable. The identically fabricated sensors show similar response and sensitivity while wash test shows the stability of sensor after washing. The developed sensor is also able to detect the moisture caused by both artificial sweat and blood serum, which will be of value in developing new sensors tomorrow for smart wound-dressing applications.

## 1. Introduction

The sensors are devices that correlate the electronic world to the physical and shows exceptional enactment for the development of new applications [1,2]. Nowadays, a variety of sensors are in use for solving routine problems. These sensors include temperature [3], light [4] and touch [5]. Some others include gas [6] employing titanium dioxide (TiO_2_) nanoparticles decorated BP nanosheets as the sensing layer to detect ammonia [7]. Humidity [8] and moisture sensors that are also able to detect exhaled gas and distance variation of moisture emitting objects [9] are also in use. In our daily life, wearable sensors have become a part of health monitoring [10,11]. With rapid progress in flexible electronics, these sensors are introducing as a new method to achieving a healthy life by real time monitoring of behavioral [12] and physiological data [13,14]. These wearable sensors are attached to the human body or clothes [15] and interact with an external or host computer to provide information about the human’s health [16] and physical conditions [17,18]. For example, pressure, friction and moisture are the most influential parameters in the formation of pressure ulcers in health monitoring [19].

Furthermore, unusual symptoms of many life-threatening diseases can be obtained from daily sweating profiles, serving as an effective quantitative index for early diagnosis [20]. The skin burns in hot and humid environments frequented by firefighters, and other personnel can also be prevented using these wearable sensors [21]. Most fluids such as urine, blood, tears and sweat are considered as biomarkers for monitoring the status of physiological health [22,23]. If we look into the composition of different biofluids, the composition of blood and sweat is related osmotically [5]. Although blood carries highly accurate information on the human body, sweat has the potential for easy, fast and noninvasive monitoring [22]. Thus, accurate knowledge of the dielectric properties of biofluids, specifically sweat supports the design of new sensors, wearable devices and smart technologies.

Numerous sensors based on various principles and substrates are available for the detection and monitoring of humidity and moisture [9,24,25,26,27]. These are capacitive, resistive (inductive) LC resonators or optical sensors. Capacitive sensors are the most often utilized type of sensor in industrial applications [28]. Between the electrodes of these sensors, a layer of hygroscopic substance serves as a dielectric medium. The material’s hygroscopicity is determined by various factors, e.g., hydrophilic functional groups and porosities for water accumulation [29]. Flexible sensors can be integrated into textiles in a variety of ways, including inkjet printing, screen printing, stamp transfer, electrospinning and dip coating or through the inclusion of conductive yarns during the production process [30,31]. Among all fabrication processes, embroidery has been identified as the most effective method for incorporating wearable sensors. This is due to the availability of production technology (industrial embroidery machines), the efficient use of conductive threads and the repeatability of the geometries and layouts required [32].

Different fabric substrates such as denim textile [24], cotton satin fabrics [33], cotton woven fabric, polyester woven fabric and medical cotton woven fabric [34] and boxer underwear made from cotton [35] can be utilized to develop different embroidered sensors [36]. Fabric substrates have been identified as a natural and convenient solution for developing wearable electrical sensors for these applications because the use of textile in human life. To design a wearable sensor, the textile impact on health should also be considered together with hygienic properties. The textile should not be composed of toxic elements, which are considered hazardous for the environment, human body, and health. While selecting fabric for the sensor, other main factors such as appearance, smoothness, long-lastingness and washability should be taken into account [37]. Thus, different kinds of fibers have been used in fabrics such as natural fibers (cotton and wool), chemical fibers such as cellulose fibers (viscose) and synthetic fibers (acryl, polyester, polyamide and polypropylene). Cotton and polyethene terephthalate are the most frequently used textile materials [38].

Both textile materials can be used to build any form of clothing; however, polyester textile production is primarily focused on sports and fitness due to its qualities, whilst cotton textile production is often focused on personal comfort and is frequently used in everyday apparel. These embroidered electrodes act as smart sensors and can be integrated into textile since they can blend with fabric. Silver based electrodes have broad-spectrum antimicrobial and antibacterial and antistatic properties for use in biomedical applications [39,40]. These threads are skin-friendly with great thermal conductivity and thermal stability. In addition to this, it is also stated in the datasheet provided by the supplier that these threads are applicable for seam positions that require antimicrobial characteristics [41,42].

Wound management is a very serious challenge for diabetic patients. Wounds are protected from further deterioration using bandages. Wounds must be protected from long-term exposure to wetting due either to water or sweat. It is, thus, desirable to have bandages that can detect the presence of moisture. This could be due either to accidental wetting or through perspiration or bleeding or puss leakage. In all cases, bandages will need to be replaced. The incorporation of a moisture sensor in the bandages will, thus, be very useful in recognizing small (few drops) volumes of bio-fluid or ordinary water. On a commercial scale, available wound sensors act as an indicator for a change in wound dressing. The WoundSense sensor is a commercially available moisture sensor. It sits directly on the dressing to find the moisture status of the wound without disturbing or removing the dressing. During application of sensor to 588 patients, 44.9% of dressings changes were unnecessary [19].

Hence, there is a need to develop a textile-based embroidered sensor that should be able to detect moisture caused by sweat or blood or both together for biomedical application where wound recovery is targeted. The desired attributes are cost-effective fabrication, impedance/admittance change with moisture, design stability, repeatability, compatibility with human skin and negligible hysteresis response. The main aim of the present research is to fabricate moisture sensors that could be validated in isolation but may be combined with bandages/dressings in biomedical applications. The sensors of tomorrow could act as smart bandages that can help in the early treatment of wounds. For a smart dressing sensor, the sensor could be applied at specific points on the body to evaluate the condition of wounds to change the dressing or drug delivery in a timely manner. In the case of its application as a sweat sensor, it will be wet with sweat once integrated as wearable sensors.

The present study proposed to develop a capacitive sensor on textile substrates using electrodes made of conductive silver threads, silver-tech 150 and HC 12. Two different silver threads were selected because of the difference in their electrical resistance. HC 12 is more conductive compared to silver-tech 150. According to the data sheet provided by supplier, HC 12 and silver-tech 150 electrical resistances are <100 Ω/m and <300 Ω/m, respectively. Threads properties are not expected to change with temperature. This is because conductive threads are used only to embroider electrodes of the IDE, and changes of electrodes conductivity will not affect actual capacitance as long as the dielectric constant of the textile has none to very low sensitivity to temperature. The moisture is expected to linearly change the dielectric constant of the substrate; thus, the said capacitive sensor, through its admittance, provides high sensitivity, low-temperature dependence, small size and small power consumption, with the possibility of sensing large varieties of physical and chemical parameters. The sample for the measurement of parameters may be in gaseous form or liquid form [43]. The capacitance will only change if properties of the fluid between electrodes changes with changes in temperature or humidity. Moreover, it is reasonable to expect that sensor temperatures remain very close to the body temperature, and it will not be exposed to direct sunlight but rather remain in contact with the skin, and cover from the other side will reduce environmental impacts and any temperature variation.

The remainder of the paper is organized as follows: Section 2 describes Material and Methods relating to fabrication of sensors, the substrate used, the electrode material properties and the measurement approach. Section 3 describes various results such as admittance vs. moisture behavior and sensitivity, repeatability, wash resilience, etc. Finally, the paper concludes with the Conclusions section.

## 2. Experimental

### 2.1. Material and Methods

#### 2.1.1. Threads and Substrate

A woven cotton fabric serves as the substrate for manufacturing the sensor. Commercial conductive threads Silver-tech 150 from Amann, Bönnigheim, Germany and HC 12 from Madeira, North Yorkshire, UK were used to embroider the sensor on the textile substrate. Silver-tech 150 is a silver-coated polyamide continuous filament with a linear density of 110 × 2 and a diameter of 0.1 mm. Other thread HC 12 is made entirely of polyamide/silver plated yarn; its linear mass density (dtex) without the silver coating is 2 dtex with a count of 235, whereas it has a 610 dtex ± 15 dtex with the silver coating, according to the datasheet provided by the company. HC 12 has a diameter of approximately 0.17 mm. The Hitachi TM3000 tabletop scanning electron microscope was used to evaluate the morphology of conductive threads and cotton substrate (SEM).

#### 2.1.2. Tested Samples Preparation

Tap water was used for moisture testing. Other samples of fluids tested in the present research were artificial sweat and blood serum. Artificial sweat of pH 6.5 was prepared according to the European standard EN1811 [44,45]. It was prepared by mixing 0.1 wt% of urea, 0.5 wt% of NaCl and 0.1 wt% of lactic acid in deionized water. The pH of artificial sweat sample was adjusted to 6.5 using 1 M NaOH, 0.1 M NaOH and 0.1 M HCl. The blood serum sample was collected in the hospital, Galetic clinic, Novi Sad, Serbia, by a certified nurse. The volunteer understood and signed the consent form to volunteer his blood sample for experimental use. The serum sample was centrifuged and processed in the hospital. The serum was stored at 4 °C in a freezer before it was used for experiment. To use in experiments, the serum sample was removed from a freezer and kept at room temperature, 25 °C.

### 2.2. Design and Fabrication of Sensors

**Figure 1:**
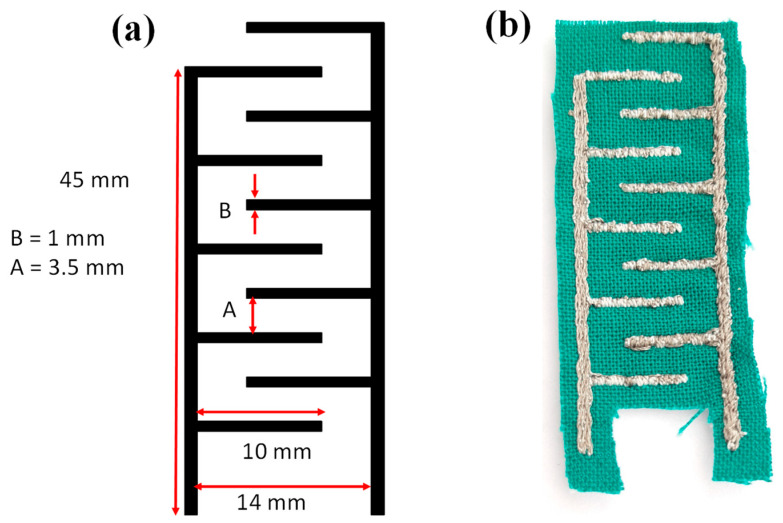
Interdigitated electrodes; (**a**) sensor layout and (**b**) embroidered sensor.

Using ZSK software provided by the ZSK machine, Krefeld, Germany, the AutoCAD sensor design was converted to an embroidered file. The stitch type and length of embroidery were tuned to ensure high-quality electrodes in terms of operation and signal consistency. The conductive thread was inserted into the needle, while the other (cotton thread) was used as a bobbin. On a textile substrate, electrodes with a finger width of 1 mm and an interspacing of 3.5 mm were embroidered using the ZSK Stickmaschinen GmbH, Krefeld, Germany embroidery machine. The proposed moisture sensor is based on the capacitive interdigitated electrodes, and the dimension of IDE is shown in Figure 1.

### 2.3. Impedance/Admittance Measurements

**Figure 2:**
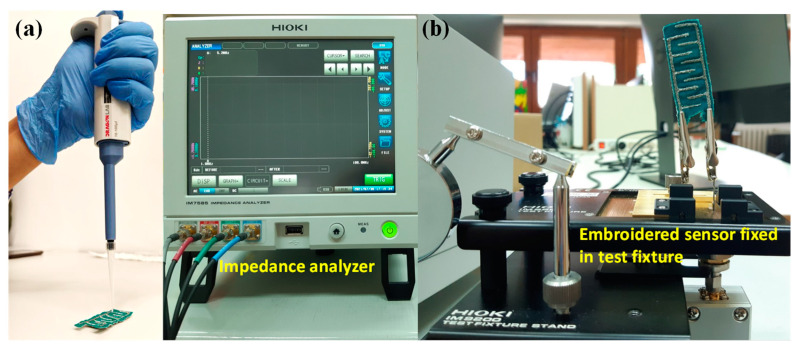
(**a**) Microliter pipette for fluid application on the sensor and (**b**) impedance analyzer with embroidered sensor fixed in test fixture IM9202 and microliter pipette.

The change in impedance of the sensor with moisture was measured with Hioki IM7585 Impedance Analyzer, Nagano, Japan at a frequency range of (1–10) MHz using normal tap water. The amplitude of the AC voltage applied for impedance measurement was 420 mV, which is the default value of the used impedance analyzer. The impedance change was measured first for dry sensors in order to obtain a reference value of the sensor’s impedance. The water was distributed between electrodes finger on fabric with microliter pipette. Water was dropped in steps from 1 to 50 µL on the sensor at the same place to observe liquid distribution throughout the sensor. All experiments were conducted at room temperature, 25 °C. The sensor response to environmental humidity was negligible since it was tested at different times of the day before proceeding with experiments with different fluids. The experimental setup for a better understanding of research methodology is shown in Figure 2. The measured impedance magnitude data were then converted to admittance magnitude to explore admittance vs. moisture behavior, as it was expected to linearly vary with moisture.

## 3. Results and Discussion

### 3.1. Morphological Analysis of Threads and Cotton Substrate

**Figure 3:**
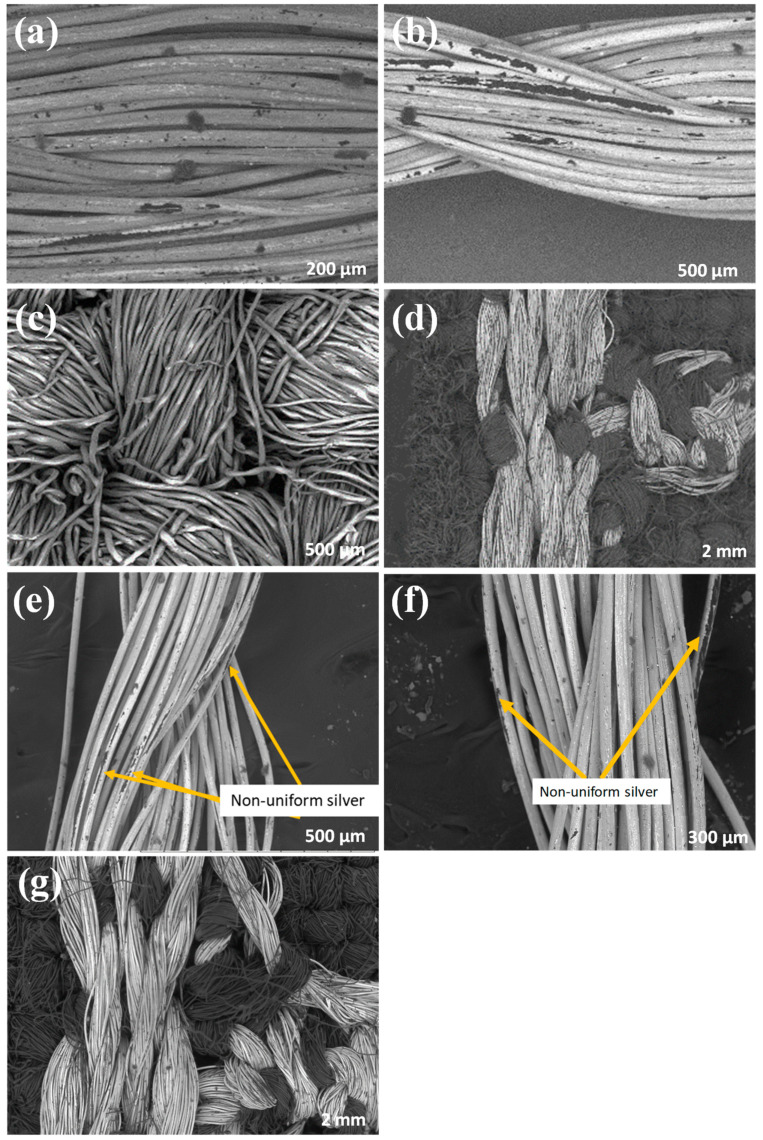
SEM analysis of threads and embroidered sensors before and after washing, respectively: (**a**) Silver-tech 150, (**b**) HC 12, (**c**) fabric substrate, (**d**) embroidered electrodes, washed (**e**) Silver-tech 150 (**f**) HC 12 and (**g**) embroidered electrodes.

Figure 3a–d represent the SEM of silver-tech 150, HC 12, textile and embroidered electrodes on textile, respectively. Figure 3e–g represents the SEM of washed thread silver-tech 150, HC 12 and embroidered electrodes. These threads are silver coated 2-ply yarns. For silver-tech 150, each ply is composed of 110 silver-coated fibers, whereas for HC 12, each ply is composed of 610 silver-plated fibers. The presence of silver coating can be seen in SEM images that appeared as the white/silver color on the threads. Figure 3c presents the woven and permeable structure of the textile. It has a higher thread count per inch than most cotton substrates, making it an excellent substrate for sensor fabrication The porous morphology of textile substrate will facilitate the buildup of liquids through the embroidered sensor.

The fabric chosen for the moisture sensor is crucial because it determines how liquid is transported from the fabric to the electrodes via capillary forces. This is mostly determined by the fabric’s wettability. Wettability is determined by the following variables: (1) surface of the fabric and (2) manufacture of fabric. On the other hand, moisture absorption is dependent on the weave type, fiber geometry, pore size distribution and fabric surface density [46]. To observe these factors, the SEM of fabric and fabric with embroidered electrodes is shown in Figure 3c,d. The textile fiber density is high, its weave type is plain and it shows porous structure. The reported wettability of this type of cotton with an average thickness of 0.27 mm is 340.4 [46]. Figure 3d,g of an embroidered sensor on the cloth demonstrates how the conductive threads are interwoven with the fabric.

### 3.2. Moisture Detection by Impedance/Admittance Measurement

**Figure 4:**
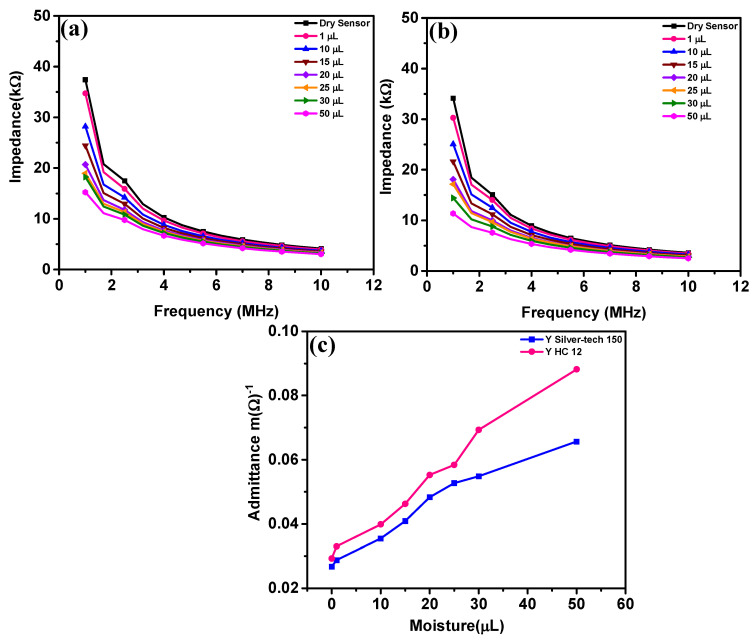
Impedance response of sensors; (**a**) Silver-tech 150 sensor with frequency, (**b**) HC 12 sensor with frequency and (**c**) admittance response of Silver-tech 150 and HC 12 sensor with different moisture volumes at 1 MHz frequency.

This section discusses the sensor’s usefulness by comparing the impedance change of two types of sensors as a function of moisture content. Due to a somewhat polarizable electric characteristic of moist cotton substrate, the embroidered electrodes exhibit significant impedance activity. It behaves as a lossy capacitor for which its dielectric constant proportionally changes to moisture while at the same time its conductivity increases as leakage sets in. To verify the sensors’ operation, an experiment was started with dry sensors at room temperature. The magnitude impedance of dry sensors embroidered with silver-tech 150 and HC 12 thread with different water volumes and frequency is shown in Figure 4. Figure 4a,b show the decrease in impedance magnitude of the sensors with frequency. Figure 4c shows the admittance response of silver-tech 150 and HC 12 sensors with moisture at 1 MHz.

The impedance drop with frequency is because, at higher frequencies, capacitor charge and discharge time becomes reduced, which results in greater flow in current through the capacitor and drop in impedance. Impedance drop with moisture content follows the same trend for both sensors. The plots for both sensors demonstrate that impedance decreases significantly at 1 MHz, but with further increase in frequency, the drop in impedance is small. This seems to indicate that impedance is inversely related to moisture. Hence, in Figure 4c, admittance versus moisture graphs are plotted at a frequency of 1 MHz to understand the workings of the sensor with a change in moisture volume. As postulated, the admittance shows linear dependence on moisture. The difference in admittance values between the two sensors is visible. The dry HC 12 sensor is nearly the same as dry silver-tech 150 sensors, whereas the admittance of the HC 12 sensor with 50 µL fluid is 40% higher than that of the silver-tech 150 sensor at 1 MHz.

Admittance increases in sensors with moisture are a result of account of increased permittivity in the system. The dielectric constant ($\varepsilon_{r}^{\prime }$) of water is 78 at 25 °C [47]. The dielectric constant of water depends on the frequency, salinity and temperature [48], but it is stable and higher than the textile dielectric constant, $\varepsilon_{r}^{\prime }$ = 1.58 [49], due to the porosity of textile substrate. When textile fabric absorbs water (water is trapped in fabric structure), it affects the electromagnetic properties of fabric by changing its dielectric constant on one hand and by leakage loss through conductive water on the other [47,50,51,52,53].

### 3.3. Characterization of Conductive Threads

**Figure 5:**
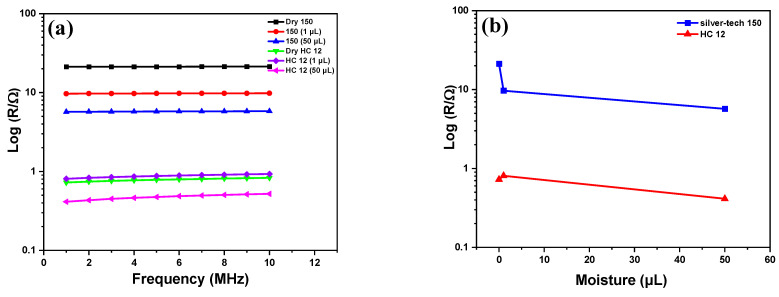
Threads response with (**a**) frequency and (**b**) moisture drop in resistance of silver-tech 150 is greater due to moisture as compared to HC 12.

Figure 3c,d represent SEM of the interlaced structure of conductive threads and substrate, which indicates that water sweeps through embroidered parts as well. Hence, in order to understand the impact of threads, we have tested silver-tech 150 and HC 12 threads with changes in frequency and moisture. The results of resistance variation are presented in Figure 5.

Conductive threads have resistive responses as a dominant character. In analyzed frequency range. The conductive threads showed very small reactance due to the parasitic effects; therefore, resistance was more important to show. Moreover, the real part of impedance (resistance) that tracked changes of impedance magnitude is shown in Figure 5. The results suggest that the impact of threads is negligible and of a second-order nature. They need to be taken into account only as contact resistance. In the model developed hereafter, we have ignored the presence of this resistance.

### 3.4. Moisture Sensor Modelling

**Figure 6:**
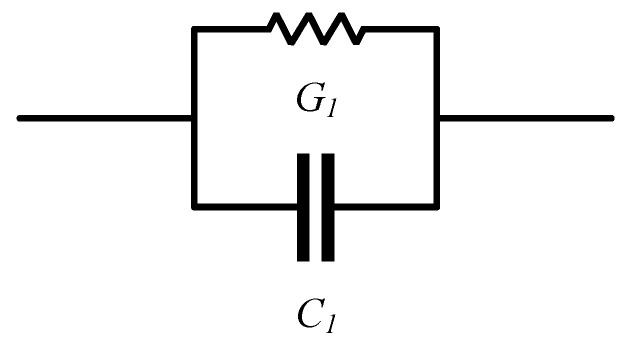
Equivalent circuit of the sensor.

The equivalent circuit of the sensor is a very useful tool for a better understanding of the sensing mechanism [54]. Our dry sensor silver-tech 150 has a capacitive nature, but it is reasonable to expect some leakage current when it is moist, since when fluid is present, there is a conductive path between some fingers of the IDE. Therefore, an equivalent circuit of our sensor can be a parallel connection of conductance *G*_1_ and capacitor *C*_1_, as shown in Figure 6.

Equivalent complex impedance *Z* of the circuit from Figure 6 is calculated using Equation (1). Figure 6 supports the equivalent electrical circuit; as with increasing frequency, there is a decrease in impedance. A parallel connection of capacitor and resistor has frequency dependence because of a change in capacitor impedance according to Equation (1).

(1) $$aZ=R+jX=\frac{1}{G_{1}}+\frac{1}{j\omega C_{1}}$$

**Figure 7:**
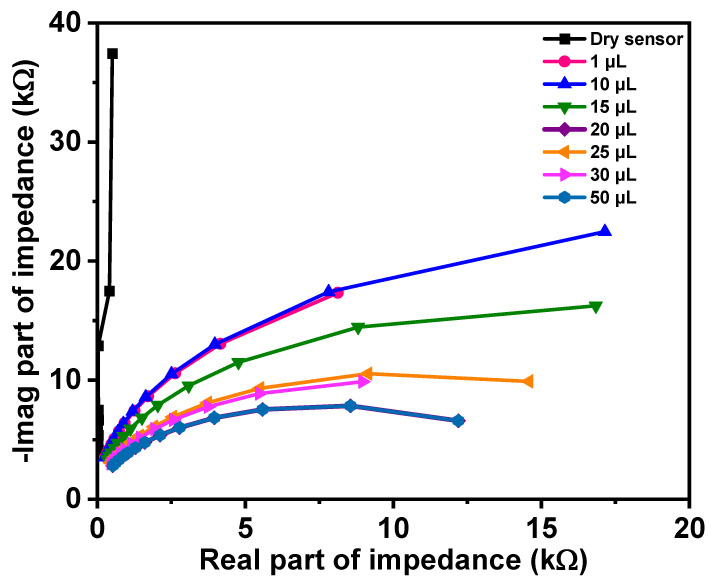
Comparison of Nyquist plots for various liquid levels.

A parallel connection of capacitor and resistor has frequency dependence because of a change in capacitor impedance according to Equation (2):

(2) $$Z=\sqrt{{\left(\frac{1}{G_{1}}\right)}^{2}+{\left(\frac{1}{\omega C_{1}}\right)}^{2}}$$

with an expected decrease as frequency increases. Therefore, from the measured real part (*R*) and imaginary part (*X*) of the sensor impedance, it is possible to calculate parameters of the equivalent circuit at each angular frequency, as shown in Equations (3) and (4):

(3) $$G_{1}\left(\omega_{i}\right)=\frac{1}{R\left(\omega_{i}\right)}$$

(4) $$C_{1}\left(\omega_{i}\right)=-\frac{1}{X\left(\omega_{i}\right)\omega_{i}}$$

where *i* is the measurement index out of *N* measurement points. Finally, unique values of *G*_1_ and *C*_1_ can be calculated as means of *G*_1_(*ω*_i_) and *C*_1_(*ω*_i_). With an increased amount of fluid between fingers, it is reasonable to expect that conductivity will increase and that permittivity will be higher as well. The dielectric constant of water is 78 [47], while for dry cotton textile is 1.58 [49]. Therefore, *G*_1_ should increase and so should *C*_1_, with increasing moisture. From Figure 7, it can be observed that the Nyquist plot of the dry sensor is a straight line that is very close to the Nyquist plot of the pure capacitor. Moreover, we can observe that there is a clear distinction between curves with increasing moisture.

In Table 1, estimated values for model parameters of dry sensor and sensor when different amounts of fluid are applied are shown. All values were calculated using Equations (3) and (4). From Nyquist plots of measured values for the dry sensor, it can be observed that it has very high resistance (range of Mega Ohms). This can also suggest that, in the case of dry sensors, a pure capacitive model can be used, as shown in Equation (5).

(5) $$Z=jX=\frac{1}{j\omega C_{1}}$$

Using means of values obtained with Equation (4), the estimated capacitance is 3.8731 pF, while the mean of values obtained using Equation (5) is 3.87 pF. Therefore, the difference is less than 0.01%, which confirms the reliability of our proposed model. It can be observed from Table 1 that, with increased fluid quantity, there is an increase in *G*_1_ and an increase in *C*_1_. This was expected, as discussed above. Please notice that the values in Table 1 are means of estimated values for *G*_1_ and *C*_1_ on frequencies in the range of 1–10 MHz.

**Figure 8:**
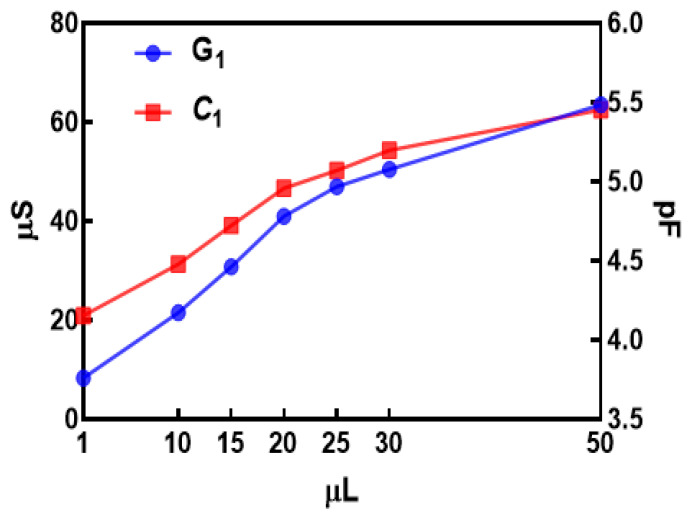
Model parameter changes with moisture level.

Graphical presentation of model parameter changes with moisture level is shown in Figure 8, indicating good linearity between conductance and capacitance with respect to moisture levels.

In addition to the plot shown in Figure 8, we calculated admittance magnitude using values of *G*_1_ and *C*_1_ from Table 1 with Equation (6).

(6) $$\left|Y\right|=\sqrt{G_{1}^{2}+{\left(\omega C_{1}\right)}^{2}}$$

**Figure 9:**
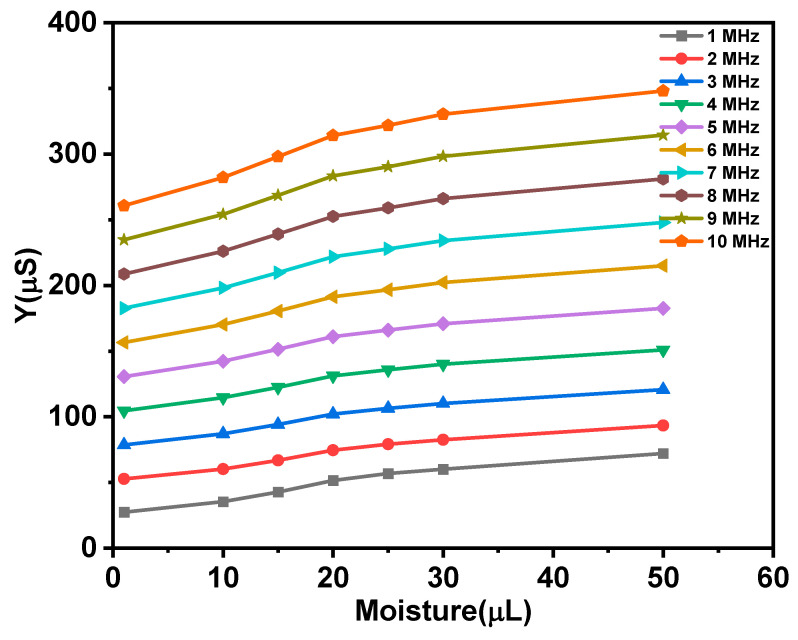
Total admittance changes with moisture level.

Graphs shown in Figure 9 indicate linearity between total admittance and moisture levels on all frequencies. Therefore, relationship admittance moisture will be used in future sensor applications.

**Figure 10:**
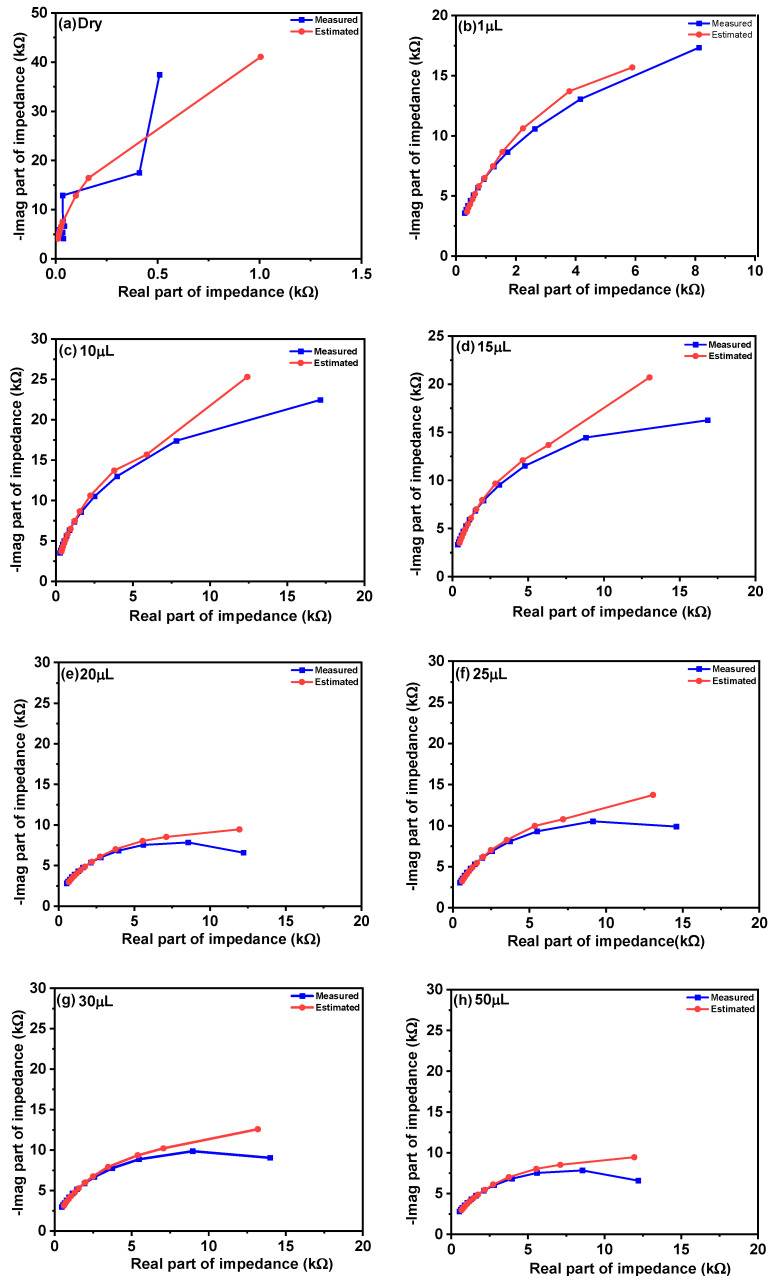
Comparison of impedance of moisture sensor for measured and estimated values at different concentrations; (**a**) dry, (**b**) 1 µL, (**c**) 10 µL, (**d**) 15 µL, (**e**) 20 µL, (**f**) 25 µL, (**g**) 30 µL and (**h**) 50 µL.

A graphical comparison (Nyquist plot) between measured and estimated values is shown in Figure 10.

### 3.5. Sensitivity Analysis of Moisture Sensor

**Figure 11:**
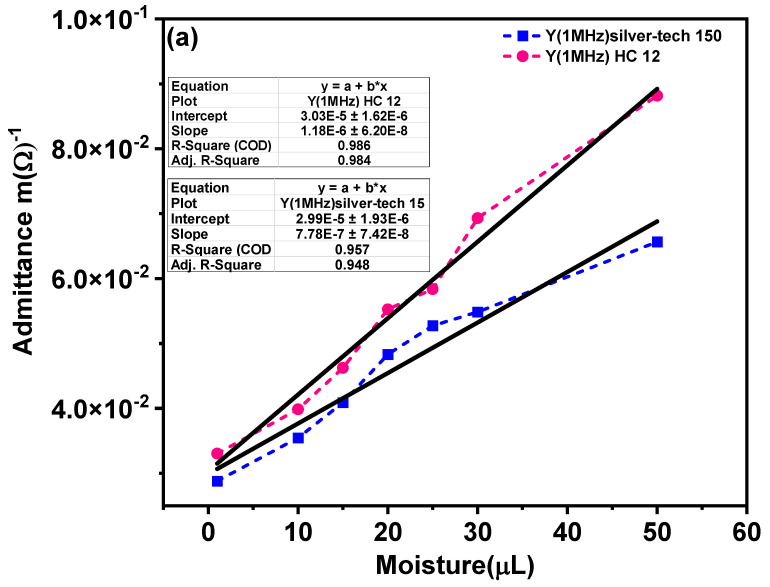

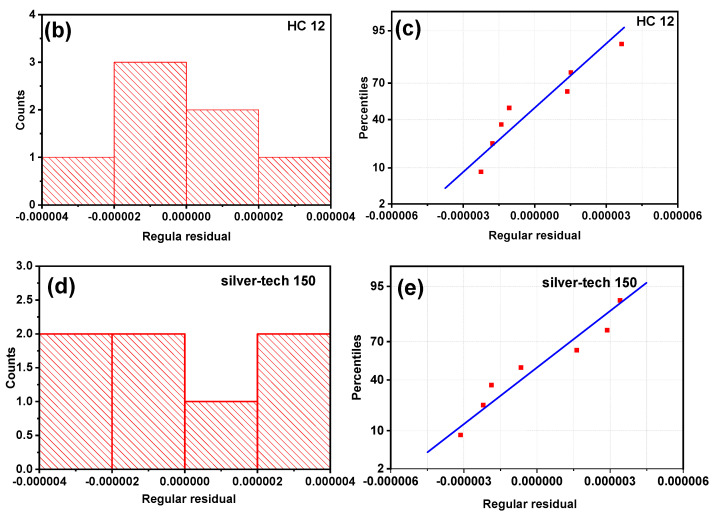
Sensitivity test of moisture sensor at 1 MHz for (**a**) sensitivity model (**b**,**c**) residual histogram and fit of HC 12 and (**d**,**e**) residual histogram and fit of silver-tech 150, respectively.

The sensitivity is an important parameter for checking the functionality of the sensor. Figure 11 represents the model for admittance change with respect to the moisture for silver-tech 150 and HC12. The fitted curve for admittance illustrates the transfer function of the moisture sensor operating at 1 MHz. In model Equation (7), *x* denotes the volume of the fluid while *y* denotes the admittance magnitude. The minimum volume used for sensors is 1 µL, arising from the fact that a micropipette was used for dropping fluid on to the sensor. The sensitivity of the sensors can be calculated from the slope of Equation (7). The sensitivity of two sensors calculated is presented in Table 2.

(7) y=a+bx

From the calculated values, it can be observed that the sensitivity of the sensors is comparable, although silver-tech 50 has slightly lower sensitivity than HC 12. The performance and sensitivity of the sensor depend on the number of fingers and the area of the contact surface of the IDE with the medium under study [43].

Increasing the area of contact increases sensor sensitivity and performance. Hence, in order to increase the sensitivity of the sensors for real-time application, the sensor area must be enough for sensitivity enhancement to detect a low volume of moisture [55]. In Figure 11b–e, we additionally present the residuals resulting from the fit. It can be observed that the residuals are equally distributed in Figure 11b,d and the fit is very good, as shown in Figure 11a.

### 3.6. Adsorption–Desorption Study of Sensors

**Figure 12:**
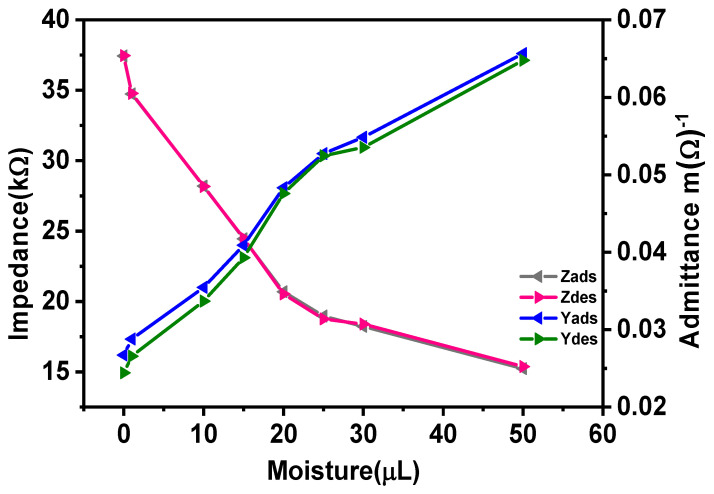
Hysteresis loop represents the adsorption–desorption response of sensors.

The hysteresis curve of the moisture sensor is a significant parameter since it shows the sensor’s reuse reliability explained in terms of the maximum time lag between the adsorption and desorption process of the sensor. Figure 12 shows the adsorption–desorption response of the silver-tech 150 sensor in terms of impedance/admittance change with moisture volume. The data for the hysteresis curve were collected at room temperature of 25 °C. Adsorption data were collected when the sensor was adsorbed with different moisture volumes, while desorption data were collected during the process of drying the sensor from 50 µL to dry condition. The adsorption process is represented by downward arrows and desorption by upward arrows. The percentage hysteresis at different volumes of water is summarized in Table 3. The percentage value of hysteresis is calculated from Equation (8) [25].

(8) $$H\;\left(\%\right)=\frac{Zi{,}_{D}-Zi{,}_{A}}{Z_{max}-Z_{min}}100\%$$

where $Z_{A}$ and $Z_{D}$ are impedance value of adsorption and desorption at particular moisture volume, whereas $Z_{\max}$ and $Z_{min}$ are the maximum and minimum values of impedance at a maximum and minimum value of moisture volume. It can be observed that percentage hysteresis at any volume is less than 1.13%, which is an extremely low value indicating no residual hysteresis effect.

### 3.7. Performance Repeatability of Identically Fabricated Sensors

**Figure 13:**
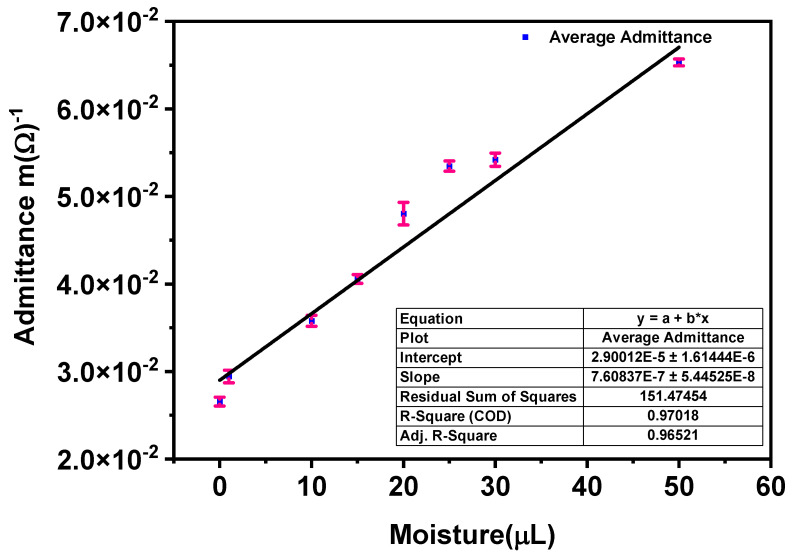
The admittance of three sensors (silver-tech 150); performance test at same fluid volume, representing the average and spread.

In wearable sensors, the repeatability of the performance of sensors over identically fabricated sensors is very desirable. In the present research, three sensors were embroidered with silver-tech 150 thread. A repeatability test was performed with these three samples of silver-tech 150 sensors. Admittance response was measured from 0 to 50 µL at 1 MHz in the present research study. Figure 13 shows the average and spread of admittance response of all the three sensors embroidered with silver-tech 150. It can be observed that all the three sensors have nearly identical admittance response. The slight variance seen in data could be explained by the uncontrollable experimental process of dropping the fluid at some location and its spread across the sensor’s surface. The variability in sensor data can also be attributed to the variability in surface and its roughness. These findings show that admittance values are influenced by the coverage of sensor surface by liquids.

### 3.8. Washing Test

**Figure 14:**
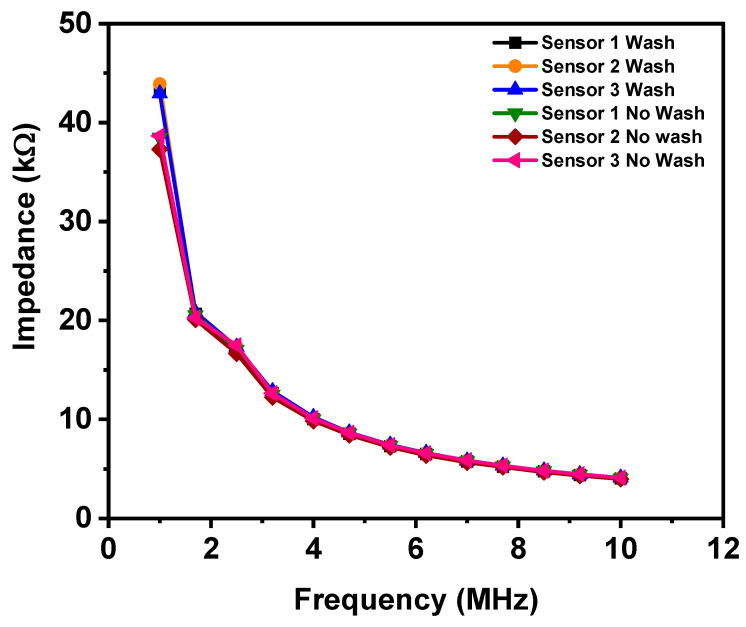
Washing and no washing test of sensors (Silver-tech 150).

Sensors must be washed before being integrated with e-textile. The test demonstrates the utility of sensors in real-world applications. Three sensors (silver-tech 150) have been washed by hand using mild detergent as recommended in the datasheet and rinsed with tap water at room temperature without considering the volume of water being used for washing. The results are presented in Figure 14. After washing and drying at room temperature, a small increase in impedance has been observed for the sensors. The reason for impedance increase could be related to water absorbed by polyamides filament through the non-uniform layer of silver coats as shown in Figure 3. As observed in SEM of threads and sensors, the silver coating is damaged. The damage to silver coating can happen due to abrasion during the packaging or embroidery process [56], which may appear as a non-uniform coating in Figure 3, SEM images.

The silver coatings are considered as uniform if the entire surface of polyamide threads is covered with an even layer of silver coating without any breakage of silver coatings. Water does not affect silver coatings because silver-coated threads are stable while washing with water according to the datasheet provided by the supplier but polyamides are affected by water through non-uniform or abrasive parts of the threads. The water molecules attach to the amide groups of polyamides, which are mainly responsible for water absorption. Hence, the dimensions of fibers made from polyamide change with the absorption of water molecules due to capillary forces [57]. Thus, the diameters of polyamide fibers alter in response to the absorption of water molecules [58], which cause defects and cracks in a silver coating, affecting the sensor’s impedance.

### 3.9. Applications of Sensor

**Figure 15:**
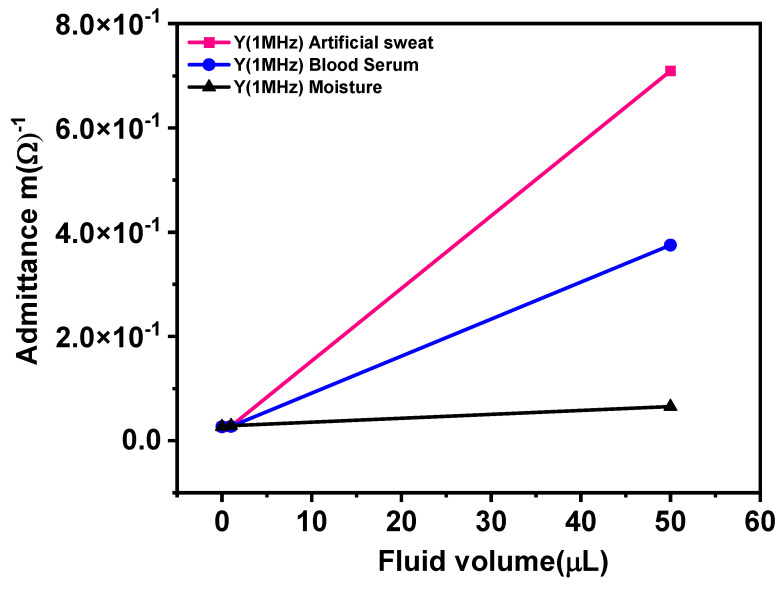
Artificial sweat and blood serum testing with Silver-tech 150 sensor at 1 MHz.

Impedance/admittance sensors work on the principle that liquids have different electrical conductivities or relative permittivity. The embroidered sensor was also tested for sweat and human blood serum. Sweat is also one of the main irritants in the wound healing process. Hence, the sensor was tested for artificial sweat and blood serum to confirm that the moisture caused by them can be also be detected. This will have important impacts on the design of a suitable sensor for smart dressing applications. In order to observe the initial working of the sensor, the artificial sweat and blood serum was tested for only two different volumes of fluids, 1 µL and 50 µL at 1 MHz, since the expected admittance behavior is linear and any two points of observation will suffice. The results of tested samples are shown in Figure 15.

The sensor response due to moisture caused by all the three fluids can be observed at 50 µL. The volume of fluids is almost at the same level but the admittance increases due to artificial sweat and blood serum are 91% and 83% as compared to moisture, respectively. Sweat is mainly composed of metabolites and minerals, dominantly Na⁺ and Cl¯. The presence of ions tends to decrease the impedance of the sensor by creating a double layer of charges at liquid sensor interface [43,55]. Human serum also contains, proteins, minerals and electrolytes. Its admittance value reported by other researchers is also in the range of millimhos at 1 MHz [59].

The results indicate that all the fluids can wet the sensor and moisture therein is detectable. Linearity is assumed on the basis that, just as the tap water and the minerals therein, the quantity of minerals and metabolites in the sweat scales linearly. Thus, the fraction of minerals/metabolites present in 1µL is same as in 50 µL. The water-based measurement proves that electrode and the substrate do not saturate with 50 µL of sweat; hence, we do not expect 50 µL of blood serum or artificial sweat to saturate sensor electrodes. For applications requiring detection of moisture due to any or mixture of fluids, the developed sensor can be used. However, in the present form of the sensor, it cannot differentiate between the types of fluids. In the future, our research will focus on investigating suitable design of sensors that can also recognize the type of fluid for smart bandage applications.

### 3.10. Moisture Sensors: Past and Present

Previous research on fabricated sensors performance for moisture detection has been summarized in Table 4 to highlight the significance of the present research. Previous research was mostly about the detection of moisture, sweat or other biofluids. These sensors are developed with some complex techniques. However, in the present case, the sensor can be fabricated with a simple approach that can detect moisture without being affected by environmental humidity.

## 4. Conclusions

The present study examined a textile-based embroidered capacitive sensor for its prospective use in wound management by detecting the presence of moisture in a bandage when the moisture could be caused by accidental wetting by water, sweating, bleeding or puss leakage. It has been shown that the sensor can detect the moisture and its quantity by changing impedance/admittance at 1 MHz for (1–50) µL moisture volume. The change in admittance magnitude happens due to a change in dielectric properties of the substrate in contact with moisture. The sensitivity of the sensor shows comparable performance for both silver-tech 150 based sensor and HC-12 based sensor. Being textile-based, it is also shown that the sensor could be reused after washing. The proposed electrical equivalent model of the sensor comprising the said capacitor in parallel with a leakage resistor was also validated. The present sensor is simple in design and does not require a complex fabrication process. Future work will focus on further exploring how best to utilize such a sensor in a smart bandage for detecting moisture and also identifying the fluids and testing them with patients.

## Figures and Tables

**Table 1:** Estimated values of model parameters.

Sample	*G*_1_ (µS)	*C*_1_ (pF)
Dry	0.6	3.9
1 (µL)	8.3	4.2
10 (µL)	21.5	4.5
15 (µL)	30.8	4.7
20 (µL)	40.9	4.9
25 (µL)	46.9	5.1
30 (µL)	50.4	5.2
50 (µL)	63.5	5.5

**Table 2:** Sensitivity of the sensors, silver-tech 150 and HC 12.

Sensor	Sensitivity (mΩ^−1^/µL)
Silver-tech 150	7.78 × 10^−7^
HC 12	1.18 × 10^−6^

**Table 3:** Hysteresis of all moisture points.

Moisture (µL)	Adsorption Impedance (kΩ)	Desorption Impedance (kΩ)	Impedance Hysteresis (%)
0	37.4	37.5	0.2
1	34.7	34.8	0.2
10	28.2	28.2	−0.1
15	24.4	24.5	0.2
20	20.7	20.5	−0.9
25	18.9	18.8	−1.1
30	18.2	18.4	1.1
50	15.2	15.4	0.7

**Table 4:** Different sensors for moisture detection.

Material	Type	Substrate	Fabrication Method	Moisture/Application	Ref
Silver paste	Impedance and transient plane source (TPS)	polyester woven fabric	Screen printing, ultrasonic spray coating	1–250 µL/Sweat	[12]
Silver-plated nylon yarn, stainless steel yarns	Resistive	Cotton/urine detection	Embroidery	5 drops of micropipette/urine	[35]
Conductive silver yarn, PVA yarn	UHF-RFID tag	Cotton	Embroidery	Water moisture/volume not mentioned	[60]
Anodic aluminum oxide (AAO)- assisted MoS_2_ honeycomb structure	Resistive		Physical vapor deposition	(0.3–0.9) L/h/Skin moisture with respect to sweat	[61]
Silver plated and coated polyamide	Admittance	Cotton	Embroidery	(1–50) µL	Present research

## Data Availability

The data presented in this study are available on request from the corresponding author.
